# Effect of family-centered care interventions on motor and neurobehavior development of very preterm infants: a protocol for systematic review

**DOI:** 10.1186/s13643-021-01612-w

**Published:** 2021-02-18

**Authors:** Manasa Kolibylu Raghupathy, Bhamini Krishna Rao, Shubha R. Nayak, Alicia J. Spittle, Shradha S. Parsekar

**Affiliations:** 1 Department of Physiotherapy, Manipal College of Health Professions, Manipal Academy of Higher Education, Manipal, 576104 India; 2 Department of Physiotherapy, University of Melbourne, Melbourne, Australia; 3 Public Health Evidence South Asia, Prasanna School of Public Health, Manipal Academy of Higher Education, Manipal, India

**Keywords:** Family-centered care, Motor development, Neurobehavior development, Very preterm infants, Systematic review

## Abstract

**Background:**

Globally, very preterm birth is a health concern leading to various developmental difficulties such as poor motor and/or cognitive function. For infants born very preterm, family-centered care (FCC) might promote developmental skills over the time in an appropriate enriched environment. The purpose of this study is to systematically review and assess the evidence of FCC interventions on the motor and neurobehavioral development in very preterm infants. Additionally, this review aims to determine the factors that might affect infant development.

**Methods:**

Systematic review will be carried out by including (a) quasi-randomized controlled trials and randomized controlled trials (b) of very preterm born infants (born < 32 weeks of gestation), and their primary caregivers will be included in the review (c) who received FCC-based interventions such as collaborative interaction between a healthcare professional and a parent, home program, home visits, and parent education, and (d) measure motor and neurobehavioral function. Electronic databases such as Scopus, PubMed, Embase, Cochrane Library, Web of Science, CINAHL, and PsycINFO will be searched using database-specific terms. Additionally, searches will be carried out in ProQuest, and references of included studies will be searched. Two review authors, independently, will conduct the screening, data extraction, and critical appraisal of included studies. If possible, a meta-analysis will be undertaken to assess the effect of the FCC on the motor and neurobehavior of premature infants.

**Conclusion:**

The review will provide insights regarding the effect of the FCC on preterm infants. This systematic review will guide the clinicians on the feasibility of practicing FCC that might support and promote the integration of parents into various rehabilitation settings.

**Systematic review registration:**

Protocol has been registered in PROSPERO on August 26, 2020.

**Supplementary Information:**

The online version contains supplementary material available at 10.1186/s13643-021-01612-w.

## Background

### Description of the condition

Infants born between 28 and 32 weeks of gestation are considered “very preterm” infants [1]. Globally, approximately 5 to 18% (15 million) of neonates are born preterm of all the live births [2]. The rate of very preterm born vary throughout the world, and in the USA, about 1.6% of live births are born as very preterm [3]. However, there is dearth of data on proportion of neonates born very preterm in other countries. These babies are at high risk of impaired cognitive, sensory, motor, emotional, language, and behavioral development when compared to full-term infants [4]. Early brain injury and impaired brain development are reasons for the aforementioned problems [5].

In the first year of life, biological, environmental, and social factors influence the development of early motor and neurobehavior development. Motor development encompasses the quality of movement, developmental milestones, motor skills, visual-spatial, visual-motor integration, balance, and coordination [6]. Neurobehavioral development occurs with efficient responses to the sensory (tactile, auditory, visual, olfactory) stimulation and autonomic nervous system, organization of state (calm, excited, irritable), and self-regulation (hand to mouth responses), language, attention, socio-emotional development, and executive function [7].

Very preterm infants are usually admitted in the neonatal intensive care units (NICU) to provide special care and management. In the NICU, very preterm infants face many stressful situations such as excessive sound, bright light, painful medical applications, and lack of parental contact, which they would not have experienced in utero [8]. Excessive sensory load on tactile, olfactory, gustatory, visual, and auditory systems during this critical period of brain development impairs baby’s physiological responses and may impact negatively on their neuromotor and behavioral development. Therefore, the relationship between preterm birth and the aforementioned chain of events might affect the development of motor skills and neurobehavior in the later span of life [9].

Family and home-based environments are considered to have the greatest and permanent effect on an infant’s growth within ecological system, even when taking into consideration of certain other aspects such as education and socioeconomic status of the parents [10]. Birth of a very preterm infant affects the mental well-being of the parents, leading to distress and anxiety, and alters the sense of bonding and parenting skills to care [11]. A review conducted to see the effects of discharge communication practices in pediatric emergency care reported parental education as an early intervention strategy that might provide a significant opportunity for improving parent comprehension along with benefits of health outcomes of children [12]. Therefore, strategies focused primarily on responsive parenting might promote the growth of the child through family dyadic relationships [13].

### Description of the intervention

Family-centered care (FCC) is described as a healthcare approach that involves planning, delivery, and evaluation centered on equally positive relationships among families, healthcare providers, and patients. FCC’s values include family care, inclusive family engagement, interaction, empathy for and integrity of families, and knowledge transformation [14]. It has been implemented in developed and a few of the developing countries [15]. FCC can be practiced in various settings such as homes, clinics, hospitals, and communities. The central focus of the FCC is to train parents as a primary therapist and provide psychosocial support [14]. FCC has shown to be a promising approach for children (0–12 years of age) by providing faster recovery, emotional/behavioral support to a child, parental satisfaction, and reduced cost due to hospitalization [16].

### How the intervention might work

Approaches such as developmental care, Newborn Developmental Care and Assessment Program (NIDCAP), and Creating Opportunities for Parent Empowerment (COPE) have components of FCC, which are shown to be beneficial on medical and developmental outcomes of preterm infants [17–20]. Interaction between an infant and parent begins before infancy and is greater at childhood, with social-emotional experiences involving gestures, cries, smiles, and mutual gaze vocalizations, and continues throughout childhood [21]. A significant aspect affecting the growth of the child is the quality of interaction between an infant and parents. Modification of an infant’s physical and emotional environment both in the NICU and post-hospitalization at home will have positive outcomes in their overall development [22, 23]. The FCC has shown an increased knowledge, capacity, and competence of the parents to care for their infant or child [24]. The potential benefit of the FCC is that it is relatively low cost and has the focal involvement of parents for a long-term period. It has been shown to decrease the length of hospitalization, improve the well-being of preterm by allowing better allocation of resources, and enhance parent-infant bonding [24, 25]. In spite of benefits, FCC may make preterm infants vulnerable to infections transmitted through parents/family members or may catch hospital-acquired infections. However, a neonatal infection can be prevented with precautionary measures such as hand hygiene, proper handling techniques, clean clothing, and environmental hygiene [26].

### Why it is important to do this review

In infants born very preterm, certain aspects of motor and neurobehavior function are frequently impaired relative to their counterparts born at term. FCC has been found to support the infant’s care by enriching the environment, improving cognitive and physical growth, and further promoting early developmental resilience (right from the birth) [27, 28]. One of the reviews described that family-based interventions reduce preterm parental stress, and it has cost-effective benefits when practiced in the NICUs [25]. There was a significant improvement in weight gain, reduced hospital readmission, and positive parental outcomes (skills of infant care; parent satisfaction; reduced anxiety, stress, and depression) among the FCC intervention group when compared to the control group [13]. However, the review [13] did not address the effect of FCC on motor and neurobehavior development among infants. Other existing reviews were on developmental care intervention, and the intervention was delivered by health professionals alone and not by involving parents [17, 18]. Additionally, these reviews were conducted on infants born at 37 or lesser weeks of gestation without subgroup of very preterm born infant. Hence, there is a need to explore the effects of FCC on motor and neurobehavior development of preterm infants.

Literature suggests that a supportive family environment, a positive bond between parent and infant, leads to improved neurodevelopmental outcomes even if the preterm infants are exposed to vulnerabilities such as neurological abnormality [28, 29]. The studies have shown that negative and intrusive parenting leads to poor developmental outcomes in preterm children across childhood. On the contrary, warm, sensitive, and positive parenting might result in a protective effect on the preterm infant’s development [30].

However, to the best of our knowledge, no systematic review has studied the impact of FCC interventions on the motor and neurobehavioral function in very preterm infants during both the NICU stay and the follow-up period. Furthermore, the various factors (mode of delivering the intervention, dosage, etc.) that might influence the success of FCC are not yet known. Hence, we would anticipate that the findings from this review will help inform clinicians, parents, and educators about the role of the FCC in promoting motor and neurobehavioral development in preterm infants. With this background, we have proposed two research questions: (1) What is the evidence of data available on the impact of FCC on very preterm infants when compared to standard care/interventions without involving a family on motor and neurobehavior development? (2) What are the factors that determine infant development due to FCC interventions?

**Fig. 1:**
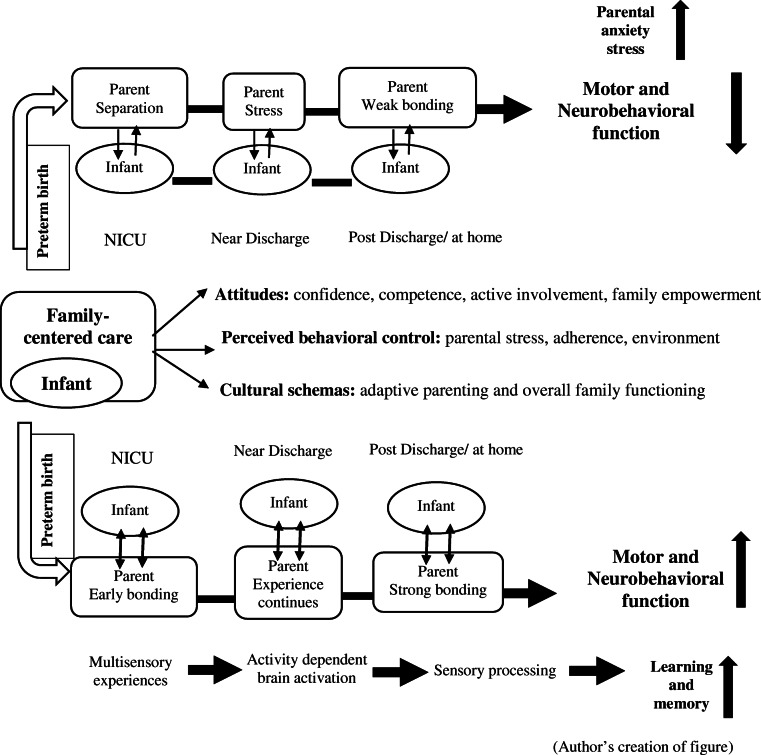
Conceptual framework of family-centered care on motor and neurobehavior development of preterm infants

We have described the relationship between the very preterm infant and FCC along with their possible outcomes through a conceptual framework (Fig. 1). In NICU, if there is limited parent-infant interaction, this might result in parental stress and poor bonding between them. This might further impact on delay or poor motor and neurobehavioral development of the infants and increases the level of parent anxiety and stress. On the other hand, involving parents or implementing the FCC right from the beginning in the NICU might create a well-nurturing environment and a positive parent-infant bonding due to the effect of various sensory experiences and activity-dependent brain activation. Further, this might accelerate the development of motor and neurobehavioral function along with the increased ability of learning and memory in preterm infants.

## Methods

### Review registration and reporting

The systematic review has been registered in PROSPERO (the International Prospective Register of Systematic Reviews) on August 26, 2020. We have adhered to PRISMA-P (Preferred Reporting Items for Systematic Review and Meta-analysis) guidelines to report this protocol (supplementary data file 1).

### Eligibility criteria

#### Types of studies

Randomized controlled trials and quasi-randomized trials having at least two groups are eligible to be included. Non-randomized trials, observational studies, study protocols, editorials, reviews, conference abstracts, letters, and commentaries will be excluded.

#### Type of participants

Preterm infants born lesser than 32 weeks of gestation and their primary caregivers. Primary caregivers can be either mother, father, or grandparents.

#### Place of recruitment

This review does not impose any restrictions on the setting. It can be in the NICU, hospital, and home or community.

#### Time of recruitment and follow-up

The studies should have recruited infants from birth in the NICU stay to 6 months, and followed up till 2 years of age.

#### Intervention

This review will include any FCC interventions involving the establishment of a collaborative relationship between the healthcare professional and the parent, mutually agreed-upon goal setting, and creating the home program by selecting therapeutic activities that focus on accomplishing family objectives, supporting the implementation of the program through home visits, parent education, and evaluating the outcomes. Interventions having at least two components of FCC will be included. The intervention will involve supervision and support from a clinician or professional such as a neonatologist, pediatrician, nurse, physiotherapist, occupational therapist, speech-language pathologists, and other rehabilitation team members.

#### Type of comparators

The studies should have compared the FCC to the therapist’s provided standard care interventions or usual care. Studies comparing a type of FCC versus another type of FCC will be included.

#### Type of exposure

Such as age, time of recruitment and follow-up, settings, FCC providers, intensity and frequency of intervention, parental behavior, responsivity, and parental satisfaction that may influence infant development.

#### Types of outcome measures

##### Primary outcomes

We will include studies that measure motor and neurobehavioral function. Outcomes assessed at any time points but up to 2 years of age will be considered.

Motor functions may include quality of movement, gross and fine motor skills, developmental milestones, visual-spatial, visual-motor integration, balance, and coordination [6]. The tools used to measure motor functions could be Prechtl’s General Movements Assessment (GMA), Test of Infant Motor Performance (TIMP), Alberta Infant Motor Scale (AIMS), Neuromotor Behavioral Assessment (NMBA), Hammersmith Infant Neurological Examination (HINE), Pediatric Evaluation of Disability Inventory (PEDI), Peabody Developmental Motor Scale (PDMS), and Bayley Scale of Infant and Toddler Development (BSID).

Neurobehavior is measured in terms of the sensory and autonomic nervous system; organization of state (calm, excited, irritable) and self-regulation (hand to mouth responses); language, attention, socio-emotional development; and executive function [7]. Neurobehavior could be measured using Assessment of Preterm Infants Behavior (APIB), Brazelton Neonatal Behavioral Assessment Scale (NBAS), Neurobehavioral Assessment of Preterm Infants (NAPI), and NICU Network Neurobehavioral Scale (NNNS).

Secondary outcomes are changes in parental behaviors or responsivity captured through videotaped interactions or observations and measured by any of the validated scales. Parental satisfaction will be measured by questionnaires and interviews. Factors such as age, time of recruitment and follow-up, settings, FCC providers, intensity, and frequency of intervention that might influence an infant’s development will be considered for the review. We will also consider potential harms or risks of FCC intervention as reported by included studies, which may include, but not limited to, adverse events related to neonate, e.g., infections, and adverse events related to parent, e.g., anxiety.

### Search methods for identification of studies

#### Electronic databases

We will search PubMed, Cochrane Central, Scopus, Embase, PsycINFO (Ovid SP), CINAHL, and Web of Science. Articles that are written in English from January 2010 to August 15, 2020, will be included. The following keywords will be used: “family-centered care,” “family-centric approach,” “preterm infants,” “motor development,” and “neurobehavior development.”

#### Searching other resources

We will search in ProQuest, ClinicalTrials.gov, EU Clinical Trials Register, *meta*Register of Controlled Trials, and the World Health Organization (WHO) International Clinical Trials Registry Platform search portal. We will contact field experts and corresponding researchers of included studies to get relevant additional information. To find related studies, we shall search for reference lists and forward citations of included studies. Identified records will be exported to EndNote X7 for data management.

### Selection of studies

**Fig. 2:**
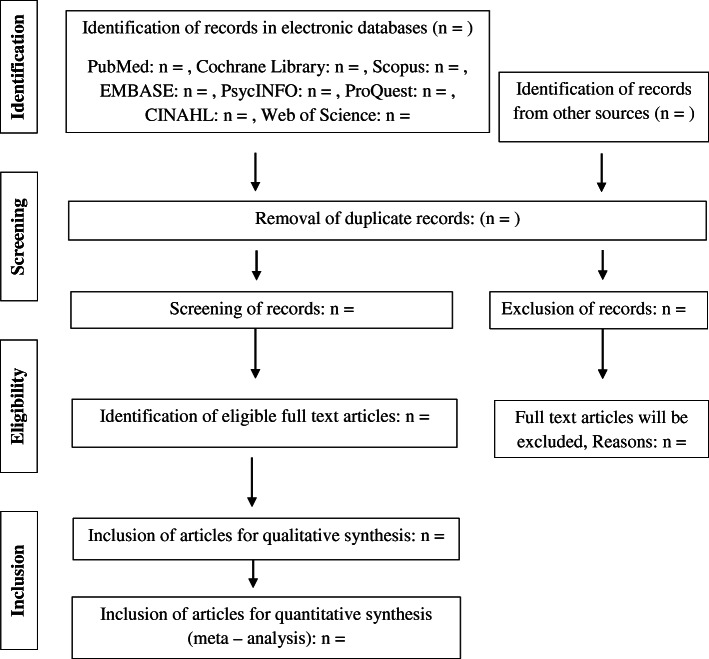
Flow chart of study selection

Two reviewers will search and read the titles and abstracts of the listed sources individually and exclude any studies depending on the eligibility criteria. If disparity arises between the authors, it will be resolved by discussions and will be reviewed as to their full text. After obtaining the full text of the included abstracts, three review authors will independently rank these as “include” or “exclude.” If necessary, we will address any differences in agreement, with a senior review author. We will record the reasons for excluding the articles. The proposed screening protocol for abstracts and full texts has been attached as supplementary data file 2. Reference details, any knowledge accessible on current research, and record details of similar publication will be considered. An adapted PRISMA will be implemented (Fig. 2).

### Data extraction and management

The extraction of general characteristics such as study identifiers, location of the study, participants, study selection criteria, and outcomes from the included studies will be carried out. The TIDier (Template for Intervention Description and Replication) checklist will be used to summarize the list of intervention characteristics and assist in the replicability of interventions and comparability between the studies [31]. Independently, two review authors will abstract the data from the articles included in the review using the data extraction templates. From each of the included studies, the following information will be extracted: study identification (title and authors), the country of study, year of publication, sample size, features of intervention, outcomes, evaluation tools, results, and conclusions. In case of disagreement, we will discuss until a consensus, or with the help of the third reviewer, any disagreements will be resolved.

### Addressing redundant and related publications

In the case of redundant papers, related records, or several primary research studies, we can optimize the data yield by gathering all the relevant details by utilizing the highly broad dataset collected through each of the documented papers. In correlation with our results, the publication which reports the longest follow-up will be considered as a priority.

### Risk of bias assessment among included studies

The Revised Cochrane Risk of Bias Tool (RoB 2.0) would be employed to critically appraise the quality of the included articles. The RoB 2.0 tool assesses the randomization process, deviations from the intended protocol, measurement of the outcome, missing outcome data, and selective reporting. Two authors will appraise all the articles, independently. There should be agreement about the existence of certain inconsistencies. The risk will be categorized into low risk, high risk, and some concern [32].

### Addressing the missing data

From the authors, missing information would be collected, and significant empirical data such as screened, randomized, intention-to-treat, as-treated, and per-protocol population will be closely analyzed. If authors will not respond within 15 days of the last communication (email), the study would be excluded from the review. We will critically appraise the concerns related to the missing data and imputation methods, e.g., last observation carried forward (LOCF). The attrition rates, e.g., drop-outs, losses to follow-up, and withdrawals, will be investigated.

### Data synthesis

The outcomes will be presented as the mean differences (MDs)/standardized mean differences (SMDs) with 95% confidence intervals (CI) unless otherwise stated for continuous variable. For dichotomous data, the effect estimate is risk ratios (RRs) with 95% CI. If there is strong evidence of homogeneous effects through findings, using a model of random effects we will mainly sum up a low probability of bias results. The meta-analysis of random effects will be viewed with due consideration of the full range of outcomes, preferably by providing an interval of predictions. In each study, the predicted range for the true treatment effect will be specified using the prediction interval. The quality of evidence of the included studies in the review will be assessed using the Grading of Recommendations Assessment, Development, and Evaluation (GRADE) system. This system grades quality of evidence at 4 levels: high (4), moderate (3), low (2), and very low (1) [33].

### Subgroup analyses and investigation of heterogeneity

In the meta-analysis, we will not record the findings of the sample as the pooled effect estimate if there is an event of major scientific, analytical, or empirical variability. Using a standard Chi-square test (significance level of *α* = 0.1), we will recognize the heterogeneity. Using the *I*^2^ statistics, inconsistency across studies will be quantified. It is considered to be a high level of inconsistency if an *I*^2^ statistic is 75% or more. If there is significant variability, we will aim to identify potential explanations for this by analyzing the features of the specific study and other subgroups. Subgroup analyses of factors such as gender, age, country, type, and mode of intervention and outcome measures (or different time points) will be carried out to explore the interaction between them.

### Sensitivity analysis

To understand how the following factors influence the effect sizes, we will perform sensitivity analyses. The factors include the risk of bias of included studies and large and long trials to understand the extent to which they influence the results. Different effect size measurements such as risk ratio and odds ratio along with various statistical models such as fixed effects and random effects models will be used to test the robustness of the results.

### Narrative synthesis

When it is not possible to conduct the meta-analysis because of significant statistical, clinical, or methodological heterogeneity, a narrative synthesis will be done. Studies would be narratively defined focusing on the intervention and outcomes. The subsequent results would be summarized using the tables and figures.

## Discussion

Through FCC, the health professionals guide the programs and work in partnership with the parents to support and guide the infant’s motor and neurobehavior development to enhance their capabilities. Providing care to the preterm infants through a family-centered approach may improve the overall infant development and in turn reduces the burden of the caregivers and enhances their capacities. The findings of this review will provide an understanding of the effectiveness of FCC components and their benefits on very preterm infants and thereby help policymakers and health professionals to adopt evidence-based decision making and practice of FCC. This systematic review will guide the clinicians on the feasibility of practicing FCC that might support and promote the integration of parents into various rehabilitation settings.

### Strengths and limitations

To the best of our knowledge, this systematic review is the first to address the effect of the FCC on improving motor and neurobehavioral outcomes in preterm infants and the factors influencing infant development. We will undertake a comprehensive search in various databases to identify the studies; however, due to resource constraint, studies published in English will be considered.

## Supplementary Information

**Additional file 1: Supplementary data file 1.** PRISMA-P checklist.

**Additional file 2: Supplementary data file 2.** Proposed screening protocol for abstracts and full texts.

## Data Availability

The data acquisition for this systematic review has not yet started. We plan to conduct the search in mid-August. Subsequently, dataset generated through this systematic review can be requested from the corresponding author.

## Abbreviations

FCC: Family-centered care
NICU: Neonatal intensive care unit
PROSPERO: The International Prospective Register of Systematic Reviews
NIDCAP: Newborn Developmental Care and Assessment Program
COPE: Creating Opportunities for Parent Empowerment
PRISMA-P: Preferred Reporting Items for Systematic Review and Meta-analysis
GMA: General Movements Assessment
TIMP: Test of Infant Motor Performance
AIMS: Alberta Infant Motor Scale
NMBA: Neuromotor behavioral assessment
HINE: Hammersmith Infant Neurological Examination
PEDI: Pediatric Evaluation of Disability Inventory
PDMS: Peabody Developmental Motor Scale
BSID: Bayley Scale of Infant and Toddler Development
APIB: Assessment of Preterm Infants Behavior
NBAS: Neonatal Behavioral Assessment Scale
NAPI: Neurobehavioral Assessment of Preterm Infants
NNNS: NICU Network Neurobehavioral Scale
TIDier checklist: Template for Intervention Description and Replication
ROB 2.0 Tool: The Revised Cochrane Risk of Bias
LOCF: Last Observation Carried Forward
WHO: World Health Organization
MD: Mean difference
SMD: Standardized mean difference
OR: Odds ratio
RR: Risk ratio
CI: Confidence interval
GRADE: Grading of Recommendations Assessment, Development and Evaluation
