# Supplementary material for: Effect of family-centered care interventions on motor and neurobehavior development of very preterm infants: a protocol for systematic review
Source: Syst Rev. 2021 Feb 18;10:59. doi: 10.1186/s13643-021-01612-w (PMC7890856; doi:10.1186/s13643-021-01612-w)
Supplement: Supplementary file 2 — Additional file 2: Supplementary data file 2. Proposed screening protocol for abstracts and full texts. [file 13643_2021_1612_MOESM2_ESM.docx]

**Proposed Screening Protocol for Abstracts**

| **SL**  **No.** | **Study design** |  |
| --- | --- | --- |
| 1A. | a. Is it a Randomized controlled trials? OR  b. Is it a Quasi-controlled trials? OR  c. Are you in doubt? | Exclude the study based on study design: if none of the options are applicable and if it is literature review, non-randomized studies, observational studies, study protocols, editorials and pre-post and study with only one group (no comparison group). |
|  | If answer to any of these above is Yes then go to point no. 2. If you are in doubt go to point no. 1B. |  |
| 1B. | a. Is it a controlled trial with two groups (intervention and control group) and have assessed the outcome at baseline and end line in both the groups? OR  b. Are you in doubt?  If answer to any of these above is Yes then go to point no. 2. |  |
| **2.** | **Intervention** |  |
|  | a. Does the study assess effectiveness of Family centered care (FCC) OR  b. Does the intervention is based on any of the FCC components such as: collaborative relationship, goal setting, parent education, home program? OR  c. Are you in doubt?  If answer to any of these above is Yes then go to point no. 3. | Exclude the study based on intervention: if any other type of intervention that does not fit in the FCC model. |
| **3.** | **Population** |  |
|  | a. Does the population under study is preterm infants <32 weeks of gestation? OR  b. Does FCC intervention involve primary caregivers: either mother, father or grandparents in the study? OR  c. Are you in doubt?  If answer to any of these above is Yes then i**nclude it for full text screening** | Exclude the study based on population: If none of the options are applicable for population. |

**Proposed Screening Protocol for Full Texts**

| **SL No.** | **Study design** |  |
| --- | --- | --- |
| 1A. | a. Is it a Randomized controlled trials? OR  b. Is it a Quasi-randomized controlled trial? | Exclude the study based on study design: if none of the options are applicable and if it is literature review, non-randomized studies, observational studies, study protocols, editorials and pre-post and study with only one group (no comparison group). |
|  | If answer to any of the above is Yes then go to point no. 2. If you are in doubt go to point no. 1 B. |  |
| 1B. | a. Is it a controlled trial with two groups (intervention and control group) and have assessed the outcome at baseline and end line in both the groups?  If answer Yes then go to point no. 2. |  |
| **2.** | **Intervention** |  |
|  | a. Does it mention explicitly FCC interventions involving a collaborative relationship between the healthcare professional and the parent, mutually agreed-upon goal setting, creating the home program by selecting therapeutic activities that focus on accomplishing family objectives, supporting the implementation of the program through home visits, parent education and evaluating the outcomes?  b. Does Interventions have at least two components of FCC approach?  c. Does intervention involve supervision and support from a clinician or professional such as a neonatologist, pediatrician, nurse, physiotherapist, occupational therapist, speech-language pathologists, and other rehabilitation team members?  d. Does studies compare FCC to the therapist provided standard care interventions or usual care?  If answer to above is Yes then go to point no. 3. If you are in doubt please mark the study for discussions | Exclude based on intervention: if any other type of intervention that does not fit in the FCC model. |
| **3.** | **Population** |  |
|  | a. Does the population under the study is very preterm infants lesser than 32 weeks of gestation? OR  b. Does FCC intervention involve primary caregivers: either mother, father or grandparents in the study?  If answer to above is Yes then go to point no. 4. | Exclude based on population if none of the options are applicable. |
| **4.** | **Outcomes** |  |
|  | a. Motor outcomes: Prechtl’s General Movements Assessment (GMA), Test of Infant Motor Performance (TIMP), Alberta Infant Motor Scale (AIMS), Neuromotor Behavioral Assessment (NMBA), Hammersmith Infant Neurological Examination (HINE), Pediatric Evaluation of Disability Inventory (PEDI), Peabody Developmental Motor Scale (PDMS) and Bayley Scale of Infant and Toddler Development (BSID).  b. Neurobehavioral outcomes: Assessment of Preterm Infants Behavior (APIB), Brazelton Neonatal Behavioral Assessment Scale (NBAS), Neurobehavioral Assessment of Preterm Infants (NAPI) and NICU Network Neurobehavioral Scale (NNNS).  c. Changes in parental behaviors or responsivity captured through videotaped interactions or observations and measured by any of the validated scales. Parental satisfaction will be measured by questionnaires and interviews.  If study has reported any of the listed outcome, include it. | Exclude study based on outcome, if none of the these mentioned outcomes are assessed |
